# Collective responses of bacteria to a local source of conflicting effectors

**DOI:** 10.1038/s41598-022-08762-4

**Published:** 2022-03-23

**Authors:** Nir Livne, Ady Vaknin

**Affiliations:** The Racah Institute of Physics, The Hebrew University, Edmond J. Safra Campus, 91904 Jerusalem, Israel

**Keywords:** Bacteria, Signal processing

## Abstract

To cope in complex environments, motile bacteria have developed a chemosensory system that integrates multiple cues and directs their motion toward regions that it deems favorable. However, we have a limited understanding of the principles that govern bacterial behavior in complex stimuli fields. Here, we followed the spatial redistribution of *E. coli* cells in perplexing environments created by a local source of both beneficial (nutrients) and hazardous (low pH or indole) effectors. We identified two fundamentally distinct collective responses: a ‘trade-off’ response, in which bacteria sharply accumulated at a distance from the source that reflected a trade-off between the propagating effectors, and a ‘bet-hedging’ response, in which part of the bacteria accumulated away from the source, avoiding the hazardous effector, while the other part evaded the repulsive force and accumulated at the source. In addition, we demonstrate that cells lacking the Tsr sensor swim toward both repellents and, surprisingly, even toward pH values well below 7. Using a numerical analysis, we could correlate the collective bacterial responses with fundamentally distinct chemotactic force fields created along the channel by the propagation of the effectors and their unique perception by the chemosensory system.

## Introduction

The ability of motile bacteria to track chemical gradients in their environment influences many aspects of their behavior, including their ability to colonize new habitats, form bacterial communities, and colonize their host^1–13^. Bacteria generically favor a higher level of beneficial effectors (attractants) and avoid potentially harmful effectors (repellents). However, in their natural environments, bacteria encounter a more complex and often perplexing chemotactic force field created by diverse sources of effectors. However, we have a limited understanding of how different signals are integrated and how bacteria cope in complex stimuli fields. A fundamental test case for the way bacteria collectively respond in a perplexing environment is their response to a local source that emits both attractant and repellent effectors^14^. The spread of opposing effectors from a local source leads to spatially correlated and opposing chemotactic force fields that provide an acute conflict. The bacterial behavior under such conditions has been previously studied by placing a capillary tube filled with both attractant and repellent in an *E*. *coli* suspension and measuring the number of cells that entered the capillary after a certain time^14^. These studies showed that the presence of an attractant in the capillary along with a repellent increased the number of cells observed in the capillary. Additional experiments using a microfluidic device found that a gradient of AI-2 formed over very short distances and times can dominate over an opposing indole gradient in controlling the bacterial distribution^15^. However, how bacteria spatially reorganize around a source of conflicting effectors is not clear.

Bacteria use large chemosensory arrays to detect multiple physicochemical signals, assess their local gradients, and integrate these signals to guide them toward a preferred direction^16–19^. The gut bacterium *E. coli* has five types of transmembrane chemoreceptors, including Tar, which responds primarily to aspartate and its nonmetabolizable analog methyl-aspartate (MeAsp), and Tsr, which responds primarily to serine. Both receptors were also implicated in mediating responses to pH and indole^20–22^. Chemoreceptors function in core signaling complexes in which two receptor heterotrimers of homodimers^23,24^, with the aid of two adapter proteins (CheW), control the activity of an associated histidine kinase (CheA)^25^. Binding interactions between CheW and CheA join core complexes to form extended receptor arrays^26–29^, which lead to enhanced cooperativity^29^ and sensitivity^30,31^ and potentially signal integration^32^. Each chemoreceptor dimer adapts to sustained stimuli through reversible methylation at a rate that also depends on its individual activity state^33,34^. Thus, in equilibrium, adaptation is restricted to the receptors that are directly stimulated, which tends to isolate the effect of different stimuli^35^. However, such an independent response might not be applicable when cells face multiple gradients simultaneously or when multiple gradients affect the same receptor. Phosphorylated CheA transfers phosphate to CheY, which, in its phosphorylated form, interacts with the switching apparatus of the flagellar motors to shift their bias from counterclockwise rotation, which produces forward swimming (run), to clockwise rotation, which produces cell reorientation (tumble). Overall, the methylation status of the receptors serves as a record of their recent stimuli history and thus enables the chemosensory system to respond to temporal changes in chemoeffector levels along the cell’s trajectory and bias its random motion in the direction that it deems favorable^36^.

Here, we studied how attractant and repellent effectors emanating from a local source dynamically reshape the bacterial distribution in the environment and how signal integration may affect the collective bacterial responses. Experiments were performed in a long, effectively semi-infinite, channel, where the concentrations of the effectors in the channel span a wide range from their maximal value at the source to zero away from it. Thus, the bacterial behavior in this setup involves their full sensory dynamic range. We specifically considered a single source that alongside amino acids (attractants) contained indole or titrated to low pH (repellents), which are prevailing effectors in the human digestive tracks^37,38^. We also restricted this study to moderate levels of these repellents, namely, above pH 4.5 and below 1 mM indole^39^. We show that despite the similar bacterial response to each of these repellents separately, when attractant was also added to the source, the bacterial behaviors were qualitatively distinct and demonstrated distinct strategies in coping with the perplexing source. The first mode of response, termed a ‘trade-off’ response, was observed with nutrients and low pH at the source. In this case, the bacteria accumulated at a certain distance from the source, effectively trading lower nutrient content for the avoidance of low and potentially hazardous pH. A distinct mode of response, termed a ‘bet-hedging’ response, was observed with nutrients and indole at the source. In this case, part of the bacterial population accumulated away from the source, optimizing the avoidance of indole, while others accumulated near the source, optimizing the utilization of nutrients. Further analysis indicated that these two behavioral modes result from fundamentally distinct force fields that may propagate from such a conflicting source.

## Results

**Figure 1:**
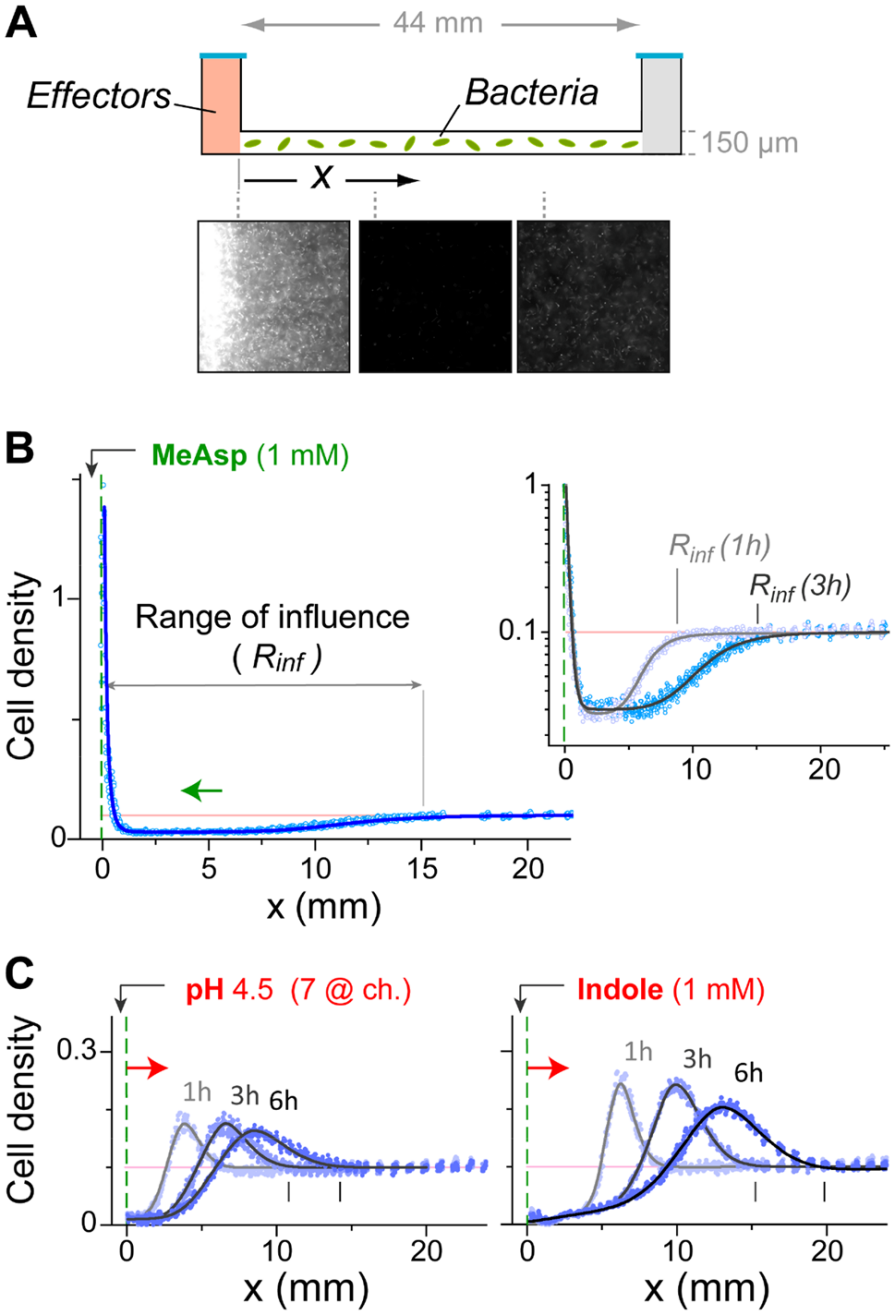
Bacterial responses to either attractant or repellent effectors at the source. (**A**) General description of the experimental setup (see also Fig. S1). A long channel was used with an effective ‘source’ on one side (left) containing the effectors. The distribution of the GFP-expressing bacteria was followed by fluorescence imaging. A few examples are also shown for an experiment with a MeAsp source. (**B**) The bacterial distribution along the channel 3 h after the addition of MeAsp (1 mM) to the source. *Inset*—the bacterial distribution at 1 and 3 h shown on a semilog scale. The ‘range of influence’ *R*_*inf*_ is defined as the distance from the source where the bacterial density approaches the initial (unperturbed) value. (**C**) The bacterial distribution along the channel at different times after the introduction of either low pH (4.5) or indole (1 mM) to the source. Throughout, cell density is normalized to the cell density at OD_600_ 1, and the initial uniform bacterial density (0.1) is marked by the light pink line.

The device used in this study consists of a long (~ 45 mm) but shallow (150 µm) channel with two reservoirs on both sides (~ 100 µl) and a bottom surface that is optically clear and oxygen permeable (Figs. 1A and S1)^30^. The channel was initially filled homogeneously with GFP-expressing *E. coli* cells. Chemoeffectors, embedded in a predialyzed agarose pad, were subsequently added to the left reservoir, which acted as a local ‘source’, and allowed to diffuse into the channel. The diffusion in the channel was generically consistent with fixed-source 1D diffusion (Fig. S1A). The channels were incubated at 30 °C, and the bacterial distribution along each channel was evaluated over time by measuring the fluorescence intensity profile and converting it to actual cell density (Fig. S1B), plotted hereafter in units of the cell density at OD_600_ 1. The bacterial density profiles were generically reproducible between replicate channels and between experiments.

### A single-effector source

We first tested each of the effectors independently (Fig. 1B,C). When an attractant effector was added to the source, the bacteria tended to accumulate near the source, and consequently, owing to the semi-infinite nature of the channel, a depletion region with reduced cell density could be clearly identified (Fig. 1A, middle image, and Fig. 1B), and expansion of this region over time could be followed (Fig. 1B, inset). The distal end of the depletion region, which is the farthest point from the source where the effector altered the bacterial distribution, is hereafter termed the ‘range of influence’, $R_{inf}$. In the case of the nonmetabolizable MeAsp effector, we could estimate the local gradient at $R_{inf}$ to be in the range of 0.1–10 nM/µm (for *D*_*MeAsp*_ in the range 7.5–8.5 × 10^−6^ cm^2^/s)^40,41^. Although the sensory sensitivity of *E. coli* cells to aspartate or serine is higher than that to MeAsp^30^, the range of influence of these metabolizable effectors was comparable to that of MeAsp, although the shape of the bacterial distribution at the far end of the depletion region was somewhat modulated (Fig. S2). An opposite response was observed when the source either was titrated to low pH or contained indole (Fig. 1C). In these cases, the bacteria clearly avoided the source and kept drifting away from it over time as the effectors diffused into the channel. The range of influence ($R_{inf}$) could be similarly defined for the repellents, corresponding in this case to the far end of the bacterial accumulation (Fig. 1C, vertical lines).

### A source containing both attractant and repellent effectors

**Figure 2:**
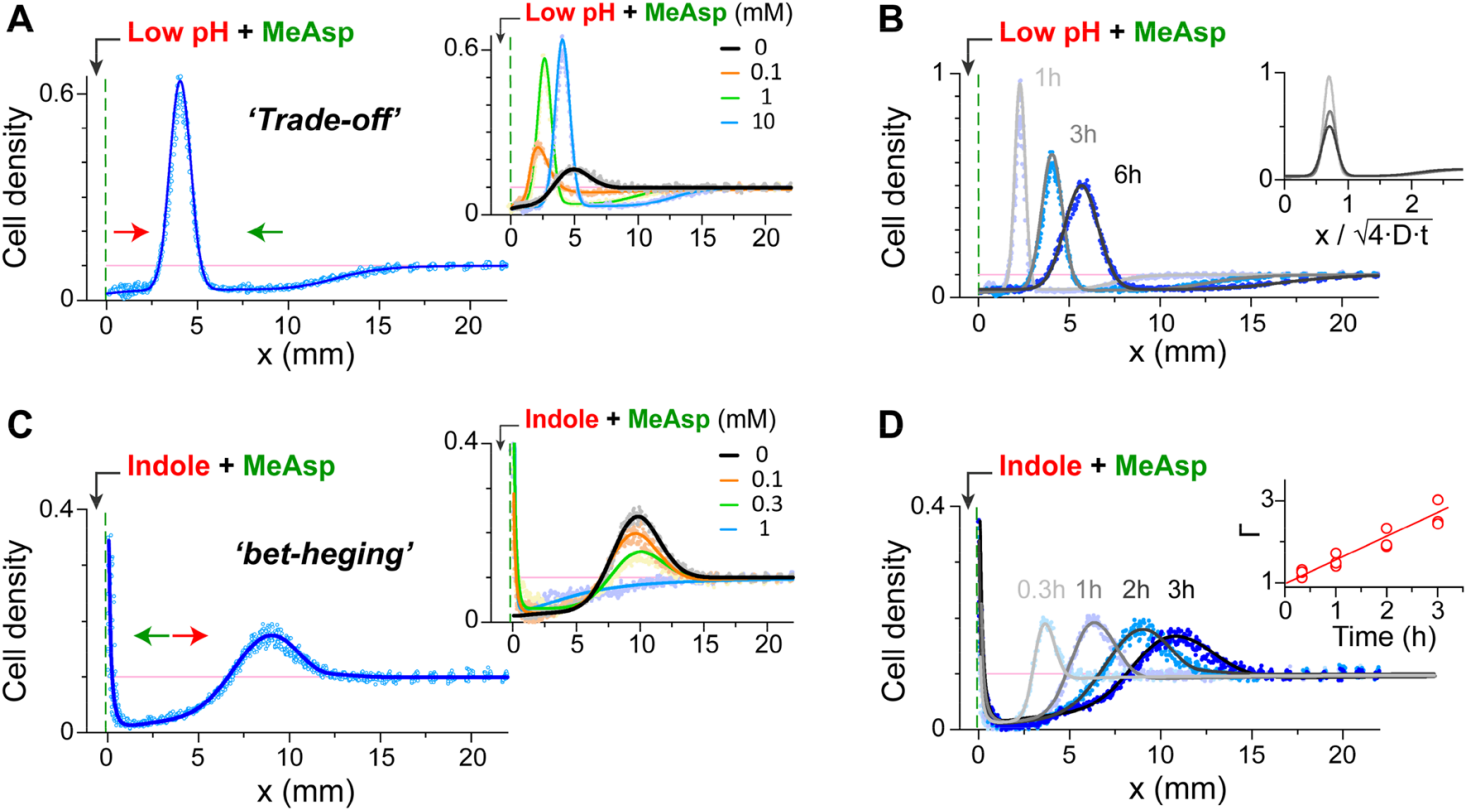
Bacterial distribution near a conflicting source containing MeAsp and either low pH (**A**, **B**) or indole (**C**, **D**). (**A**) The bacterial distribution along the channel 3 h after the introduction of a low-pH (4.5) and MeAsp (10 mM) source. *Inset*—the dependence of the bacterial distribution on the MeAsp concentration in the source. (**B**) The time evolution of the bacterial distribution in the presence of the low-pH (4.5) and MeAsp (10 mM) source. *Inset*—the bacterial distributions at various times (lines) replotted as a function of the distance from the source (*x*) divided by the corresponding diffusion distance (*D* = 7.5 × 10^–6^ cm^2^/s). (**C**) The bacterial distribution along the channel 3 h after the introduction of an indole (1 mM) and MeAsp (0.3 mM) source. *Inset*—the dependence of the bacterial distribution on the MeAsp concentration at the source. (**D**) The time evolution of the bacterial distribution in the presence of the indole (1 mM) and MeAsp (0.3 mM) source. *Inset*—the integrated bacterial population at the source (integrated fluorescence intensity, in arbitrary units) plotted as a function of time. Throughout, cell density is normalized to the cell density at OD_600_ 1, and the initial uniform bacterial density (0.1) is marked by the light pink line.

To test how bacteria cope with a source that releases both attractant and repellent effectors, we followed the redistribution of the bacteria in the channel when increasing amounts of attractant (MeAsp) were added to the source alongside either low pH (Fig. 2A,B) or indole (Fig. 2C,D). Interestingly, the addition of attractant did not simply reduce the effective repulsion of the bacteria from the source, as previously suggested^14^, but rather led to two qualitatively distinct modes of bacterial spatial organization. With low pH at the source, the addition of attractant led to a sharp accumulation of the bacteria at a certain distance from the source (Fig. 2A). Thus, in this case, the opposing chemotactic forces restricted the bacterial population to an intermediate position that reflected a ‘trade-off’ between the effectors: the bacteria were clearly drawn toward the source by the attractant, but at the same time decisively avoided the low-pH region near the source. Interestingly, the bacterial accumulation shifted away from the source at higher MeAsp concentrations (Fig. 2A, inset), suggesting that MeAsp interferes with the bacterial response to low pH (discussed below). The bacterial accumulation was dynamic, and as the effectors propagated into the channel, the bacteria slowly drifted away from the source with a speed proportional to the square root of time (Fig. 2B). We estimated that in all cases, the local pH at the position where the bacteria accumulated was above 5.2. Thus, overall, bacteria traded the lower nutrient content away from the source for a higher pH.

The response of the bacteria to a source of low pH and MeAsp was generically robust to moderate changes in the pH profile (Fig. S3). In the absence of phosphate buffer in the medium, the pH profile along the channel was approximately consistent with the generic 1D diffusion profile and independent of the presence of MeAsp. However, the presence of bacteria in the channel tended to elevate the pH and enhance the pH gradient closer to the source. Although the bacteria in these experiments were not fluorescent, they exhibited accumulation similar to that described above, as qualitatively judged by transmitted-light microscopy. As expected, the modulation of the pH by the bacteria became more moderate as the buffering capacity of the medium increased, but elevating the buffering capacity of the medium, by itself, tended to enhance the pH gradient near the source. Evidently, the bacterial spatial organization in the channel was qualitatively independent of these changes (Fig. S3).

A qualitatively distinct bacterial response was observed when indole was added to the source alongside MeAsp (Fig. 2C,D). In this case, the addition of moderate attractant concentrations to the source promoted a bipolar bacterial distribution, with cells accumulating partly away from the source, fully optimizing for the indole signal, and partly near the source, fully optimizing for the MeAsp signal. Additionally, in contrast to the pH case, in which high attractant concentrations shifted the bacteria away from the source, in the presence of indole at the source, the attractant became dominant at higher concentrations, and the overall bacterial response effectively became an attractant-like response (Fig. 2C, inset). The bacterial redistribution near the indole and MeAsp source was again dynamic (Fig. 2D). Interestingly, in this case, while the distal bacterial accumulation shifted away from the source over time, the bacterial population near the source also grew over time (Fig. 2D, inset).

**Figure 3:**
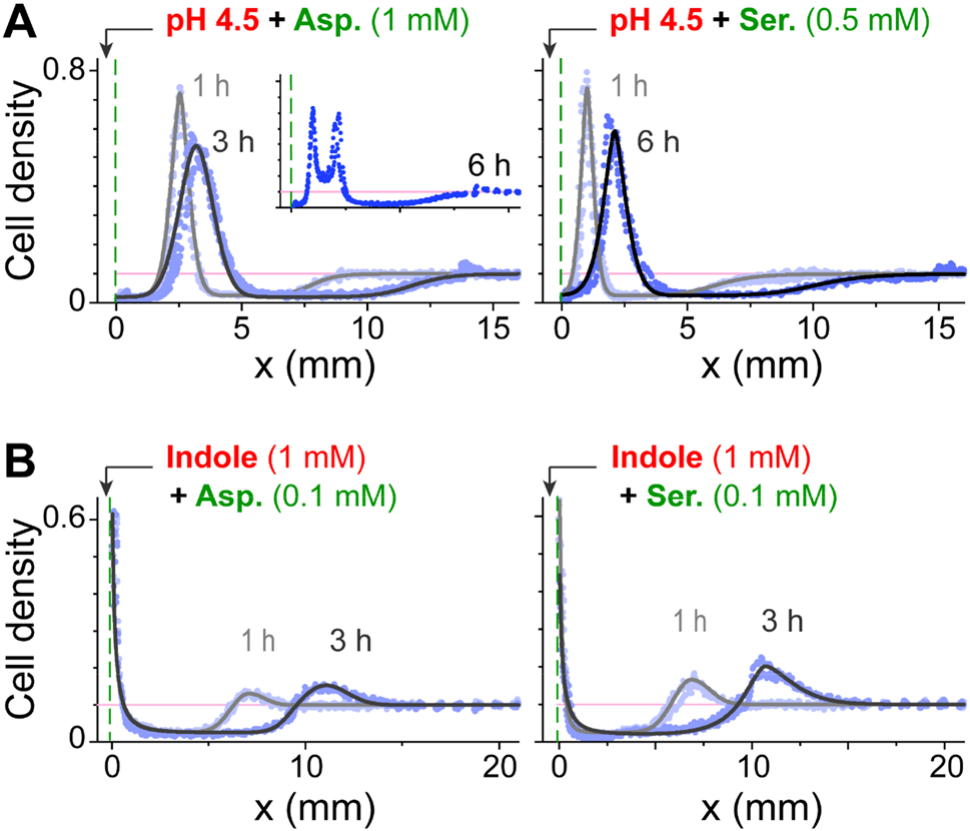
Bacterial distribution near a conflicting source that contained metabolizable attractants. The bacterial distribution measured at various times after the introduction of a source containing either low pH (**A**) or indole (**B**) along with either aspartate (left) or serine (right). *Inset* (upper left plot)—the bacterial distribution along the channel 6 h after the introduction of the low-pH and aspartate source. Throughout, cell density is normalized to the cell density at OD_600_ 1, and the initial uniform bacterial density (0.1) is marked by the light pink line.

Similar modes of bacterial reorganization were also observed when aspartate or serine, rather than MeAsp, served as an attractant at the source (Fig. 3). Up to approximately 3 h, the observed behaviors with either aspartate or serine at the source were qualitatively similar to those observed with MeAsp at the source. However, at longer times, with low pH and aspartate at the source, the accumulated bacterial population spontaneously split (Fig. 3A, inset). Since aspartate can be degraded by the bacteria, the sharp bacterial accumulation is likely to locally enhance the attractant gradient^42^ that, together with the repelling force, may split the population. Notably, however, such splitting was not observed when serine was used as an attractant (Fig. 3A, right plot). However, in the case of serine, the bacterial accumulation was generically closer to the source, possibly owing to more efficient degradation of serine by the bacteria.

To assess how the chemotaxis ability of the cells was affected by the potentially hazardous repellents, we followed the response of the bacteria to a MeAsp source in a uniformly distributed background of indole or low pH. Evidently, the chemotactic response to MeAsp was hardly affected by low pH down to 4.8, nor was it affected by indole up to 1 mM (Fig. S4A). Similarly, the presence of 1 mM indole at the source also did not prevent the bacteria from accumulating at the source when either serine or aspartate was also added (Fig. 3). Notably, however, when cells were uniformly subjected to pH values below approximately 4.65, the florescence of the cells was considerably reduced, suggesting that the regulation of the intracellular pH became less efficient, which provides a strong incentive for the bacteria to avoid these pH values. However, this effect did not have a significant influence on the observations made here (see Fig. S4 and related text), most likely because the pH near the source was somewhat elevated by the bacteria (Fig. S3B) or possibly because the very restricted low pH region made it more tolerable. Evidently, the generic bacterial accumulation observed with low-pH and MeAsp at the source was similarly observed when the pH of the source was set to 4.8 (Fig. S4B). Thus, we concluded that the observed bacterial behaviors are not directly related to a potential harmful influence of the repellents.

### The response of ΔTar and ΔTsr cells to indole or low pH

**Figure 4:**
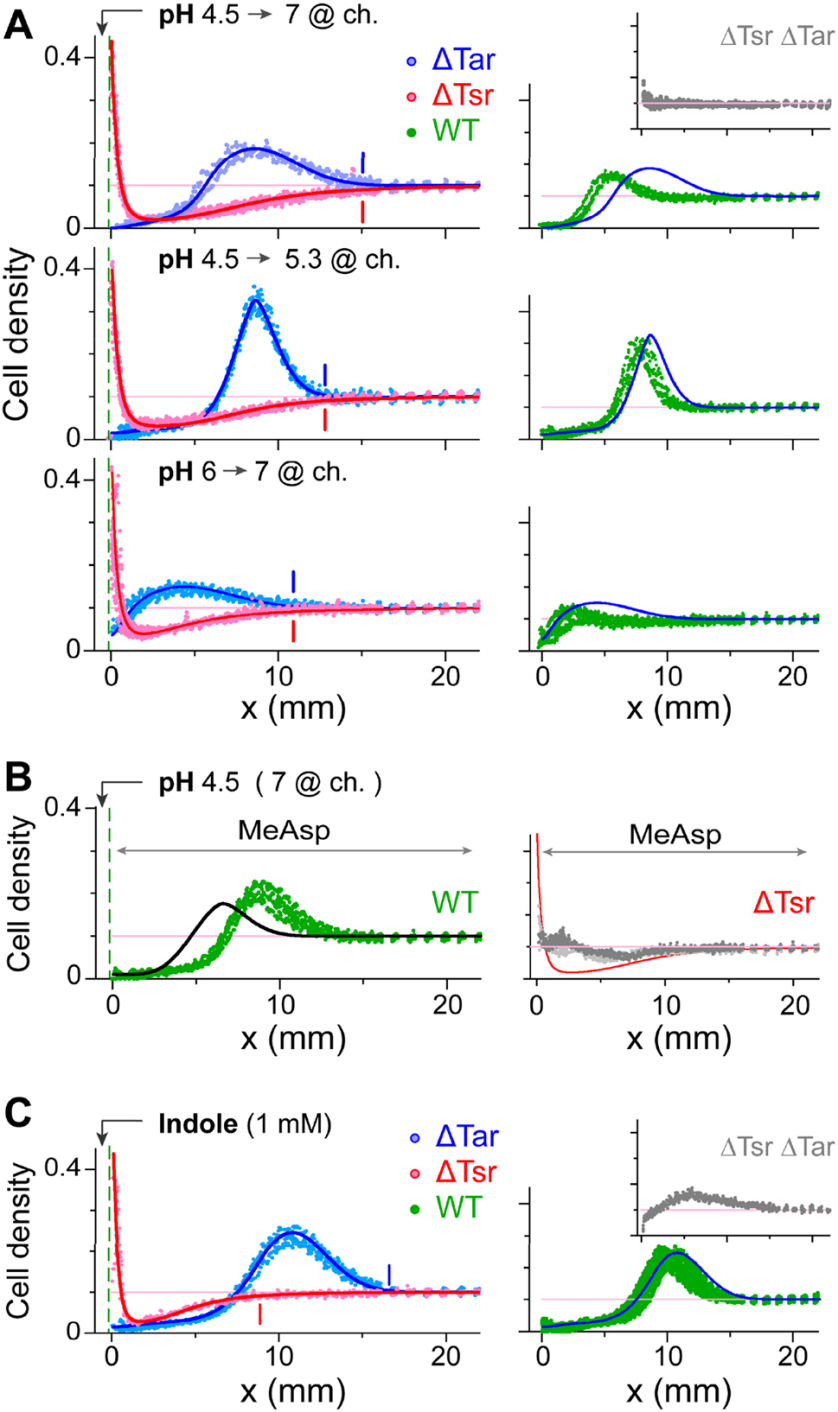
The behavior of mutant cells lacking either Tar or Tsr. (**A**) The distribution of ΔTar (blue symbols), ΔTsr (red symbols), or wild type (green symbols, right plots) cells along the channel 3 h after the introduction of a low-pH source. Data are shown for different pH values at the source and in the channel, as labeled. To allow direct comparison, the fit to the ΔTar data is also shown in the right plots (blue lines). *Inset*—the distribution of cells lacking both receptors. (**B**) *Left plot*—the distribution of wild-type cells 3 h after the introduction of a low-pH source in the presence of uniformly distributed MeAsp (10 mM; green symbols). To allow direct comparison, the fit to the corresponding bacterial distribution in the absence of the MeAsp background is also shown (black line). *Right plot*—the distribution of ΔTsr cells in similar experiments at 1 h and 3 h (light and dark grey, respectively). (**C**) As in (A, upper part) but with indole (1 mM) added to the source instead of low pH. Throughout, cell density is normalized to the cell density at OD_600_ 1, and the initial uniform bacterial density (0.1) is marked by the light pink line.

The bacterial behaviors observed in Figs. 2 and 3 depended mostly on the identity of the repellent effector rather than the attractant. Previous measurements of sensory responses to either pH^20^ or indole^21^ implicated both Tar and Tsr in mediating these responses and in creating a ‘push–pull’ balance between the signaling tendencies of these sensors^43^. Thus, we further studied the bacterial behavioral response to these repellents by testing the response of ΔTar or ΔTsr cells to either low pH or indole (Fig. 4). As expected, ΔTar (Tsr^+^) cells were repelled from low pH (Fig. 4A, blue symbols), while ΔTsr (Tar^+^) cells were attracted to low pH (Fig. 4A, red symbols), and no response was observed with cells deleted for both Tar and Tsr (Fig. 4A, inset). Moreover, the Tsr-mediated repellent response was markedly enhanced in the low pH range (pH ~ 5) compared with the higher (6–7) pH range (Fig. 4A, blue symbols). Surprisingly, the ΔTsr (Tar^+^) cells also showed clear attraction to low pH even far below pH 7 and down to pH ~ 5. The range of influence of a low-pH source was generically similar for either ΔTsr or ΔTar cells (vertical lines), further indicating that both sensors can promote chemotactic responses over a similar range of pH values. Consequently, the repellent response of wild-type cells (Tsr^+^ Tar^+^) was significantly more limited than that of ΔTar(Tsr^+^) cells (Fig. 4A, right plots). Moreover, adaptation to MeAsp tended to diminish the Tar-mediated attractant response to low pH (Fig. 4B, right plot), and correspondingly, tended to enhance the bacterial repellent response to low pH and expand its range of influence (Fig. 4B, left plot). Thus, overall, the Tar sensor is clearly effective at low pH and hinders the overall repellent response below pH 7.

Similarly, with indole at the source, ΔTar (Tsr^+^) cells were again repelled from the source, while ΔTsr (Tar^+^) cells were attracted to the source, and only a residual response was observed with cells in which both receptors were deleted (Fig. 4C). However, in contrast to the low-pH case, the attractive influence of indole (measured with ΔTsr cells) was significantly limited in range and was restricted to higher concentrations compared with the repulsive influence of indole (measured with ΔTar cells), which extended significantly further into the channel.

### Analysis of the chemotactic force fields

We further evaluated the underlying chemotactic force fields that may account for the observed bacterial behaviors. We first established a quantitative theoretical relation between the chemotactic force field $F_{che} \left( {x,t} \right)$ and the expected bacterial distribution along the channel $n\left( {x,t} \right)$. The procedure used here is detailed in the Supplementary text and briefly described below. Using this relation, we could then test to what extent the unique properties of the repellents presented in Figs. 4 and S3 and their expected influence on the force field can indeed bring about the behavior observed in Fig. 2.

**Figure 5:**
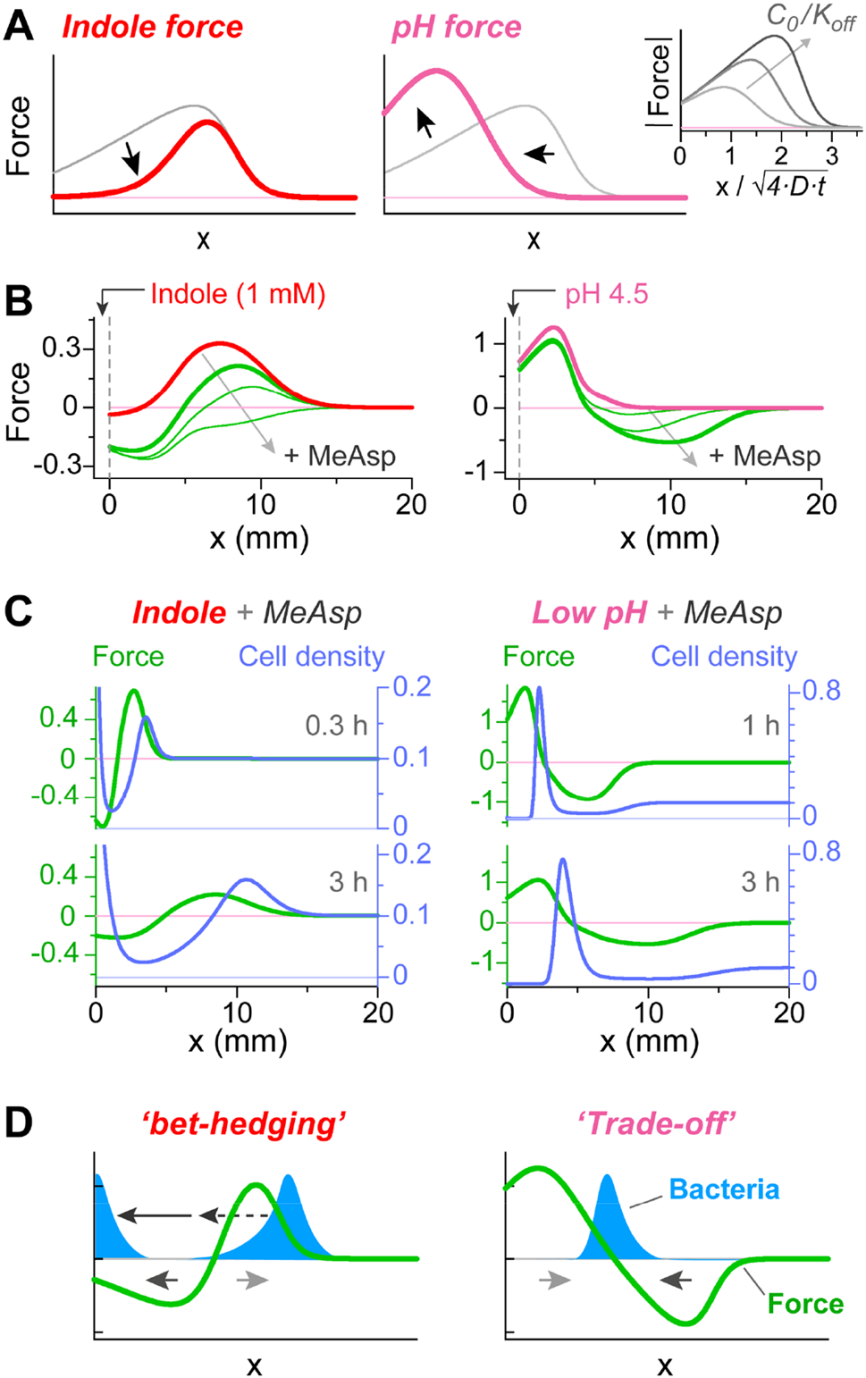
Analysis of the expected chemotactic force fields along the channel and the corresponding bacterial distributions. (**A**) Schematic depiction of the qualitative influence that the properties revealed in Fig. 4 are expected to have on the force profile, compared with its generic form (gray line). *Inset*—the force profile (in absolute value) corresponding to Eq. 1 with *K*_*off*_ being 1, 10, and 100 µM (*K*_*on*_ = 10 mM; *C*_*0*_ = 1 mM) (**B**) The force profiles obtained from the simulations by fitting the bacterial distributions (see Supplementary text and Fig. S5). Force profiles are shown for indole (left plot, 1 mM) or low pH (right plot, 4.5–7), without MeAsp (red and magenta lines, respectively) or with increasing amount of MeAsp at the source (green lines): 0.1, 0.3, and 1 mM (left plot) or 0.1, 1, and 10 mM (right plot). The MeAsp force have here a negative sign. (**C**) Force profiles (green lines, left scale) and the corresponding bacterial distributions (blue lines, right scale); the left plot corresponds to indole (1 mM) and MeAsp (0.3 mM), and the right plot corresponds to low pH (4.5–7) and MeAsp (10 mM). (**D**) Schematic illustration of the relations between the force and bacterial accumulation, based on (C).

To establish a relation between the force field and the expected bacterial distribution, we numerically integrated over time the expected bacterial redistributions using the following equations:

$$\frac{{\partial n\left( {x,t} \right)}}{\partial t} = - \frac{{\partial J\left( {x,t} \right)}}{\partial x}\quad where\quad J\left( {x,t} \right) = - D_{cells} \frac{{\partial n\left( {x,t} \right)}}{\partial x} + v_{D} \left( {x,t} \right) \cdot n\left( {x,t} \right)$$

where $D_{cells}$ is the effective diffusion coefficient of the cells due to random swimming, and $v_{D} \left( {x,t} \right)$ is the local bacterial drift velocity, induced by the local chemotaxis force. We further assumed that the contribution of each effector gradient to the chemotaxis force field can be evaluated as^42,44,45^ (see Supplementary text for more percice formulation).

1 $$v_{D} \left( {x,t} \right) \sim F_{che} \left( {x,t} \right) \sim l_{run} \cdot \frac{1}{{C\left( {x,t} \right)}}\frac{{\partial C\left( {x,t} \right)}}{\partial x}/ \left( {1 + \frac{{K_{off} }}{{C\left( {x,t} \right)}} + \frac{{C\left( {x,t} \right)}}{{K_{on} }}} \right)$$

where *K*_*off/on*_ is the effective dissociation constant of the effector for the receptor in the *‘off’* or *‘on’* signaling states, and *C*(*x,t*) is the effector spatial distribution. The distribution of a nondegradable effector can be further evaluated based on the canonical 1D diffusion (Fig. S1). The resulting force profiles are shown in Fig. 5A (inset). Notably, the force decays sharply at a certain distance that determines its range of influence (*R*_*inf*_) and depends on the diffusion coefficient of the effector and the ratio between the effector concentration at the source (*C*_*0*_) and the *K*_*off*_ of the corresponding receptor. This type of force profile can be expected for example in the case of the Tar-mediated MeAsp response (with a negative sign). In principle, the response to repellent effectors is expected to have a similar form, except for *K*_*off*_ and *K*_*on*_ exchanging their roles^46^ (see Supplementary Text).

The chemotaxis force corresponding to either indole or low pH ought to capture also their properties described in Fig. 4 and S3. Specifically, the Tar-mediated attractive response to indole would tend to reduce the repulsive chemotactic force close to the source and thus shift its bias toward the distal region (Fig. 5A, left plot). In contrast, the repulsive response to pH was considerably enhanced at low pH, both inherently (Fig. 4A) and through enhancement of the pH gradient near the source (Fig. S3). On the other hand, Tar substantially limited the overall range of influence of the pH gradients. Taken together, these properties would bias the pH force toward the proximal region (Fig. 5A, right plot). By implementing these effects in the force model, we could generate force fields (shown in Fig. 5B) that, on the one hand, obey the general properties described above and, on the other hand, generate bacterial distributions that are quantitatively similar to those shown in Fig. 2 (see Fig. S5 and related text for details).

Combining the distinct indole and low pH force fields (Fig. 5B, red and magenta lines, respectively) with the attractant force created fundamentally distinct force fields (Fig. 5B, green lines). For both repellents, the combined repellent and attractant forces had a repulsive and attractive lobe that appeared, however, in an opposite order (Fig. 5B, bold green lines). In the case of indole, an attractive lobe at the proximal region was followed by a repulsive lobe at the distal region, and vice versa in the case of low pH. Consistent with the observations in Fig. 2A,C (insets), while the overall profile of the indole force became attractant-like when combined with high concentrations of attractant at the source, the profile of the pH force field was robust. This asymmetry is directly related to the fact that, as the attractant concentration at the source is elevated, the corresponding attractant force is unchanged in the proximal region but considerably enhanced in the distal region (Fig. 5A, inset). Examples of the time evolution of the chemotactic force fields and their corresponding bacterial distributions are shown in Fig. 5C (green and blue lines, respectively). Clearly, the distinct force fields (green lines) can generate bacterial responses (blue lines) similar to those seen in the experiments. The relations between the distinct force fields and the distinct bacterial distributions are further schematically depicted in Fig. 5D.

Moreover, the force fields shown in Fig. 5B quantitatively account for further observations. The attractive part of the force generated bacterial distributions consistent with those observed with only MeAsp at the source (Fig. S5). The distinct repulsive components of the force generated bacterial distributions consistent with those observed with only Indole or low pH at the source (Fig. S5). The effective indole force (Fig. 5B, red line) could be further separated into the attractive and repulsive components that each, in turn, generates a behavior similar to that observed with the ΔTsr or ΔTar cells, respectively (Fig. S6). The enhancement of the response to pH gradients at the low pH range (Fig. 4) can also be accounted for (Fig. S7). Finally, by adding an implicit dependence of the repulsive pH force on the local ambient MeAsp concentration, which was independently demonstrated in Fig. 4B, the pH force field could also account for the shift in the position of the bacterial accumulation with increasing MeAsp concentrations (Fig. S8). However, the effective pH force (Fig. 5B, magenta line) could not be represented as a linear addition of the counter Tar/Tsr forces while demanding that each component still generates the behaviors observed with the ΔTsr and ΔTar cells. Thus, the force fields shown in Fig. 5B, C, with their distinct biases not only account to the main bacterial behaviors (Fig. 2) but also capture both qualitatively and quantitatively the sensory properties of the repellents (Fig. 4), their propagation in the channel (Fig. S3) and the interference between them (Figs. 2A, inset, and S8).

## Discussion

A basic setting for studying bacterial behaviors in perplexing environments is that created by a local source that emits both attractant and repellent effectors. In contrast to the force fields created by spatially separated sources of attractants^47^, in which each source can potentially dominate its immediate environment, at least on short time scales, a single source that emits conflicting effectors produces opposing force fields that are spatially correlated and overlapping over time and thus creates an acute conflict. Moreover, in effectively semi-infinite settings, such as the one used here, the effector concentrations at any time span the entire range from their maximal value at the source to zero away from the source, and thus, the overall bacterial behaviors involve the entire dynamic range of the chemosensory system. We specifically followed the bacterial redistribution near a source that contained amino acids (attractants) alongside either low pH or indole (repellents), which are prevailing effectors in the human gut. We show that although both low pH and indole triggered similar bacterial redistributions when present alone in the source (Fig. 1C), when attractant was also added to the source, the resulting bacterial distributions were qualitatively distinct and represented fundamentally distinct strategies for coping with the conflicting source (Fig. 2). Similar generic responses were also observed when the metabolizable serine or aspartate was used as attractant (Fig. 3). These distinct collective responses lead to semistable and structured bacterial organizations that, in turn, may affect the bacterial colonization and selective advantage in that environment.

We further demonstrated that the bacterial responses shown in Fig. 2 are consistent with fundamentally distinct force fields that propagate from the perplexing source (Figs. 5B–D and S5). These distinct force fields, in turn, are further consistent with the measured properties of the repellents and the attractants separately (Figs. S5–S8). Several factors may contribute to the enhancement of the pH force near the source: the inherently enhanced response to pH gradients at low pH (Fig. 4A), the restriction of its overall range-of-influence by the opposing response mediated by Tar (Fig. 4), the enhancement of the pH gradient itself near the source (Fig. S3), and the interference with the aspartate signal (Figs. 4B and S8). On the other hand, Tar-mediated inhibition of the response to indole at high concentrations (near the source) and possibly a more efficient spreading of indole in the channel, would lead to enhancement of the Indole force away from the source. Overall, the numerical analysis presented here demonstrates that these unique properties of the repellents can lead to the distinct bacterial collective responses observed in Fig. 2.

### A trade-off response to low-pH and nutrients

When attractant was added to a source that was also set to low pH, the bacteria tended to accumulate closer to the source but at the same time avoided the vicinity of the low pH source (Figs. 2A,B and 3). Overall, the bacteria traded nutrients loss for higher pH, while ultimately, escaping pH values below approximately 5.2. This mode of collective bacterial response is caused by an expanding chemotactic force field where the repellent force is dominant in the proximal region and the attractant force is dominant in the distal region (Fig. 5C,D, right plots). Interestingly, this force field led to a sharp and semistationary bacterial accumulation at a distance from the source that propagated at a rate proportional to *t*^1/2^ (Fig. 2B, inset).

A strong and semi-stable bacterial accumulation can readily alter its local environment. For example, the strong bacterial accumulation that was observed with low pH and either serine or aspartate at the source (Fig. 3) could be expected to alter their local environment by degrading the attractants. Indeed, spontaneous splitting of the bacterial population was observed when aspartate was used as an attractant, most likely due to local degradation of the aspartate that would produce additional opposing gradients away from the peak in both directions (Fig. 3A, inset). However, such behavior was not observed with serine at the source, possibly owing to differences in metabolism and/or sensory sensitivity that positioned the peak bacterial accumulation significantly closer to the source. Self-induced gradients are generically known to cause propagating bacterial bands^30,42,48,49^. However, these freely propagating bacterial bands were not observed to split spontaneously, suggesting the underlying opposing forces in this case are essential for such a split to occur. Note that the overall opposing repellent and attractant forces can be expected to restrict the propagating bacterial band. Another example is the ability of the accumulated bacteria to alter their local pH (Fig. S3), which, under growth conditions, could also affect the local bacterial growth rate and thus create positive feedback that further enhances the local bacterial accumulation. Strong bacterial accumulation might also trigger the formation of bacterial communities located at a relatively optimized distance from the source^50^.

### A bet-hedging response to indole and nutrients: an effective selective barrier for bacteria

When attractant was added to the source alongside indole, it had little effect on the position of the distal bacterial accumulation but instead led to bifurcation of the bacterial population, with part of the population accumulating near the source benefiting from the nutrients and the other part accumulating away from the source in compliance with the indole signal (Figs. 2C,D and 3). This mode of bacterial redistribution is caused by an expanding force field where the attractant force is dominant in the proximal region and the repellent force is dominant in the distal region (Fig. 5C,D, left plots). Interestingly, the propagation of this force field acts as a selective barrier to the bacteria. Since the force field generically propagates from the source and expands into the channel, the bacteria that reach the source must have effectively evaded the leading repulsion lobe of the force, and only after crossing this repulsion region could they be attracted toward the source. Therefore, the expansion of this force field into the channel acts as a dynamic selective barrier whose width and height change over time. The ability of the numerical analysis to capture this effect without considering bacterial heterogeneity indicates that the selection process can be based on random diffusion that broadens the bacterial accumulation in the distal region. However, in real bacterial populations, broadening of the cell distribution may also reflect bacterial variability^51^. Thus, cells that approach the source can exhibit selective traits, e.g., receptor content^52^.

The bacterial redistribution triggered by the attractant-indole force (Fig. 5C,D, left plots) is also more sensitive to the time evolution of the force. To illustrate this point, we assume that the evolution of the force profile is much slower than the bacterial chemotactic response. Since the distal repulsive lobe of the force dynamically sweeps the bacteria away from the source, if bacteria were to move by a transient perturbation to the left of this repulsive lobe, they would drift toward the source rather than away from it, and the original distribution would not be recovered. On the other hand, in the case of attractant and low-pH (Fig. 5C,D, right plots), the temporal bacterial distribution is independent of the history of the force. If the bacterial distribution were to be transiently altered, the bacteria, by following their local force along the channel, would accumulate again at the same location. These distinct properties can affect the sensitivity of the bacterial distribution to transient perturbations by other cues or by bacterial growth.

Interestingly, when bacteria grow in a confined space rich with amino acids (including tryptophan), over time, the nutrients are depleted and indole accumulates. Thus, a bet-hedging response implies that as long as the nutrient level is high and the indole level is low, bacteria in the environment are attracted to the source. However, as the nutrient level decreases and the indole level rises, cells in the environment start to be repelled by the source, although part of the population still arrive at the source and can utilize the nutrients, and only when the nutrients at the source are exhausted are the bacteria in the environment fully repelled from the source.

## Methods

### Strains and plasmids

We used the *E. coli* MG1655 (VF6) strain^30^ and derivatives with in-frame deletion of *tar* (NL1), *tsr* (MK20), or *tsr tar* (MK22). Cells were transformed with a plasmid carrying GFP under an IPTG inducible promoter (pSA11, Amp^R^, induced with 75 µM IPTG). Cells were grown over night in Tryptone broth (TB: tryptone extract 10 g/l, and 5 g/l NaCl), diluted 100-fold into fresh TB medium supplements with antibiotics and inducers and allowed to grow aerobically at 33.5 °C. Cells were harvested at an OD_600_ of 0.5 by centrifugation and washed very gently twice in motility buffer, to minimize damage to the flagella.

### Channel assay

The device used in this study was identical to the one used in Ref.^30^ (Ibidi, µ-Slide), but a different preparation protocol was employed. Here, channels were initially filled homogenously with bacterial suspension (OD_600_ = 0.1) in a commonly used motility medium that supports long-term bacterial motility but not growth, unless otherwise specified: 1 mM potassium phosphate, 10 mM sodium lactate, 5 g/l sodium chloride, 0.1 mM potassium EDTA, and 1 µM methionine, pH 7.0. Bacterial growth was negligible in this medium. One reservoir was then sealed with 1.5% agarose and covered with parafilm to prevent dehydration. Then, a second semisolid agarose gel containing the desired effectors was inserted into the other reservoir and covered. These agarose gels were premade in a special cylindrical mold and dialyzed twice: once in clean buffer to remove contamination and once in buffer containing the effectors (16 h overall). Several channels were made for each condition and placed at 30 °C in humidity chambers. The bacterial distribution in each channel was measured at various times by briefly mounting the channels onto an inverted Nikon Ti microscope equipped with a 20× (0.5 NA) or 4× (0.2 NA) objective and a decoded controlled stage and ‘perfect-focus’ system (set to the middle of the channel). The bacterial distribution along the channel was extracted from the images, after removing the measured background intensity, and normalized to the intensity corresponding to a homogeneously filled bacterial suspension at OD_600_ 1.

### Characterization of the diffusion in the channels

To estimate the general nature of diffusion in these channels, we performed experiments similar to those described above except that instead of effectors, we used fluorescein (10 µM). The spreading of this fluorophore in the channel is shown in Fig. S1 and was generically consistent with 1D constant-source diffusion (see also Ref.^30^).

### Estimating the pH gradients

To estimate the pH profiles along the channel, we used pH-sensitive fluorescein (Fig. S3). In this case, fluorescein (10 µM) was distributed homogeneously in the channel with or without cells (lacking GFP), the source was set to pH 4.5, and the fluorescence distribution in the channel was followed over time and converted to pH values by separately calibrating the fluorescence intensity to the ambient pH values. The results were independent of the dye concentration.

### Simulations

See Supplementary Text.

## Supplementary Information

Supplementary Information.
