# Supplementary material for: Collective responses of bacteria to a local source of conflicting effectors
Source: Sci Rep. 2022 Mar 23;12:4928. doi: 10.1038/s41598-022-08762-4 (PMC8943191; doi:10.1038/s41598-022-08762-4)
Supplement: Supplementary file 1 — Supplementary Information. [file 41598_2022_8762_MOESM1_ESM.pdf]

Supplementary Text: Numerical analysis

To analyze the bacterial behavior in the channel we used

$$\frac{\partial n(x,t)}{\partial t} = -\frac{\partial J(x,t)}{\partial x} \qquad where \qquad J(x,t) = -D_{cells}\frac{\partial n(x,t)}{\partial x} + v_D \cdot n(x,t)$$

where $n(x,t)$ is the cell density, D_{cells} is the cells' effective diffusion coefficient, and v_D is their chemotaxis-induced drift-velocity modeled as[42, 44, 45]

$$v_D = \alpha \cdot F_{che}\,(x,t)$$

$$F_{che}(x,t) = \ell_{run}\left(\frac{\partial f_{att}}{\partial x} + \frac{\partial f_{rep}}{\partial x}\right) \quad where \quad f_{att/rep} = \ln\left(\frac{c_{att/rep} + k_{att/rep}^{off}}{c_{att/rep} + k_{att/rep}^{on}}\right)$$

and thus, for each effector

$$F_{che}(x,t) = \ell_{run}\frac{1}{c(x,t)}\frac{\partial c(x,t)}{\partial x} \cdot \frac{\left(k_{on} - k_{off}\right)}{c(x,t) + \left(k_{on} + k_{off}\right) + k_{on} \cdot k_{off}/c(x,t)}$$

where k_{on} and k_{off} being the dissociation constant of the ligand to the respective receptor in its on or off state, and $c(x,t)$ is the ligand concentration. We also introduced ℓ_{run} to allow using a dimensionless force F_{che} (l_{run} was set to $10\mu m$), and thus, α, the chemotaxis coefficient, has dimensions of velocity.

To integrate the advection-diffusion model numerically, we set Δx to be small enough to capture features seen in the experiments (usually set to $\Delta x = 10\mu m$), and set Δt dynamically as explained below. The simulations were initiated with *n(x, t=0)* = 0.1 (homogeneous distribution), and in each time step, we calculated the effector concentrations along the channel (generically assumed to be a 1D diffusion, see Fig. S1), calculated $\mathrm{F}_{chem.}(x,t)$, and then numerically calculated the density flux as:

$$J(x,t) = -D_{cells} \cdot \frac{\partial n(x,t)}{\partial x} + \alpha \cdot F_{che}(x,t) \cdot n(x,t) \qquad with \qquad J(0) = J(L) = 0$$

where *L* is the length of the channel.

We then numerically calculated the new cell distribution as:

$$n(x,t+\Delta t) = n(x,t) - \frac{\partial J}{\partial x} \cdot \Delta t$$

while requiring $\Delta t \ll \min\left\{(\Delta x^2/ D_{cells})\,,\ \left|n/\,\frac{\partial J}{\partial x}\right|\right\}$ and verifying convergence by ensuring that the simulation's output remains unchanged upon further reduction of Δt.

Chemical profiles are quasi-static for local chemotaxis. Because the effector distribution in the channel is dynamic, when a cell swims along the channel it experiences:

$$\Delta c(x,t) = \Delta t\left(\frac{dc}{dt}\right) = \Delta t\left(\frac{\partial c}{\partial t} + \frac{\partial c}{\partial x}\frac{dx}{dt}\right) = \frac{\partial c}{\partial t}\Delta t + v\frac{\partial c}{\partial x}\Delta t \qquad (1)$$

Where v is the swimming speed of the cell ($\sim 20\ \mu m/sec$). The first term has been neglected in the simulations. This term can be evaluated as follows.

Since $c(x,t) = f(x/\sqrt{4Dt})$, the ratio between the two terms in Eq. 1 is

$$\left(\frac{\partial c}{\partial t}\right) / \left(v\frac{\partial c}{\partial x}\right) \sim \frac{x}{2vt} \qquad (2)$$

To estimate this ratio, we plotted in the figure below the range-of-influence, $R_{inf}(t)$, of MeAsp as an upper limit for x (symbols) and the expression $2vt$ (line). Clearly, $x\ /\ 2vt \ll 1$ for duration of most of the experiment.

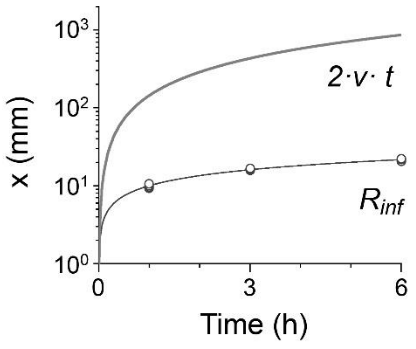

A comparison between R_{inf} (symbols) and *2vt* (line) at various times. The values of R_{inf} were taken from an experiment in which the source contained MeAsp (10mM). The value of 2vt was estimated assuming *v* = 20 μm/sec.

Correction of the cells' diffusion drift near the source. The channel was generically treated in the simulations as a 1D system. However, when cells accumulate at the source, e.g., in the presence of attractants, they tend to spread around the curved boundary of the source (Fig. S1). Since the curved boundary of the source is larger than the cross-section of the channel (its width), a band of cells that propagates towards the source is continuously expanding and thus, effectively diluted such that the cell density near the source is reduced (see Fig. S1A and related text). Moreover, since the effect is continuously enhanced as the cells approaches the source, this purely geometrical effect may modulate not just the bacterial-density at the source, but also the gradient of the bacterial density profile near the source and therefore, reduce the bacterial diffusive drift. We attempted to compensate for this effect by modifying the diffusion current very close to the source as follows:

$$J_D = D_{cells} \cdot g(x) \cdot dn/dx \quad where \quad g(x) = 0.5 \cdot \left\{ 1 + \frac{1}{1 + \left(\frac{l}{x}\right)^p} \right\}$$

with $p = 5$, $l = 2$ mm

This correction did not affect the qualitative outcome of the simulations, but in cases where bacteria accumulate directly at the source, this correction led to somewhat better description of the bacterial distribution close to the source, as seen in the figure below.

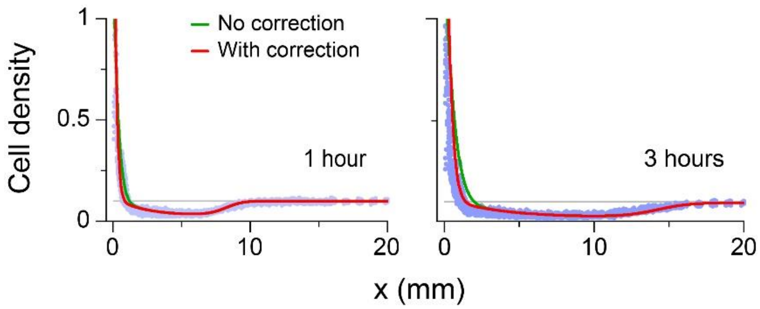

Simulation outcomes with (red line) or without (green line) the correction accounting for the modified cell gradient near the source. Also shown are the corresponding measured profiles (blue symbols). Measurements were done with MeAsp (10mM) at the source.

Supplementary: **Figure S1**

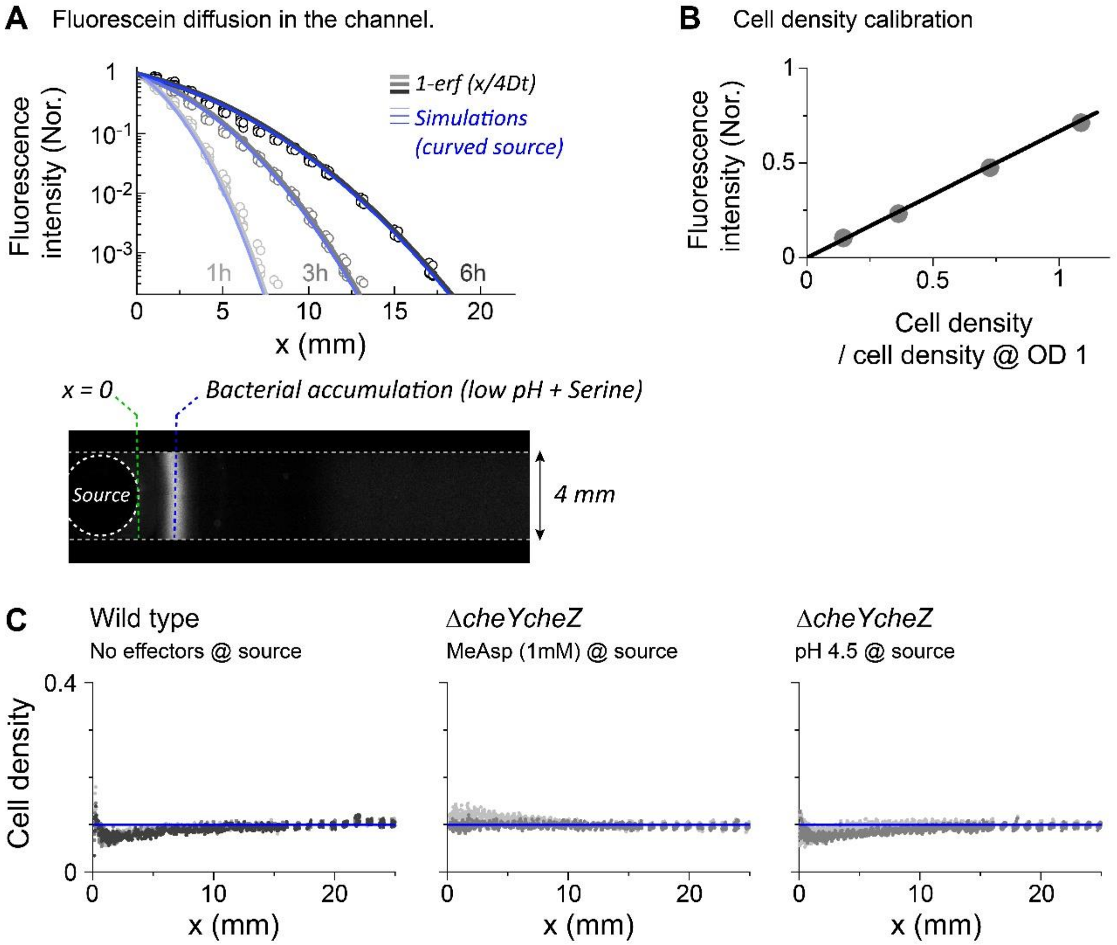

Fig. S1. Characterization of the channel assay**.**

(**A**) The diffusion profile in the channel. Fluorescein (10 µM) was added to the source and the florescence intensity along the channel was measured at various times, as labeled. Two sets of fits are shown, which mostly overlap: one, using a generic 1D constant-source diffusion: $I = I_0 \cdot [1 - \mathrm{erf}(x/\sqrt{4 \cdot D \cdot t})]$ with D=0.56·10^{-5} cm^2/s (gray lines), and second, 2D simulations of the diffusion (blue lines) considering the semi-circular shape of the source demonstrated in the image below. Evidently, the shape of the source has a negligible effect on the diffusion at the relevant time scales. Also shown is an image exemplifying the bacterial accumulation, taken 1 hour after introducing a source of low-pH (4.5) and serine (1mM). The geometry of the channel and the source are also shown. The Notably, even at a short distance from the source the bacterial band is virtually perpendicular to the channel's axis. However, when cells accumulated directly at the source interface they spread over the curved source, and thus, lower the cell density near the source. To account for this reduction, we corrected the cell density very close to the source as follows:

$$OD(x) \rightarrow \{\pi/2 - (\pi/2 - 1)/(1 + (L/x)^H)\} \cdot OD(x)$$ with L=1 mm and H=5.

(**B**) Calibration of the fluorescence intensity with respect to bacterial cell density. Suspensions of GFP-expressing bacteria with different cell densities were prepared by serial dilution, injected into the channel, and the fluorescence intensity was measured. Evidently, the fluorescence intensity in the channel was linear with cell density.

(**C**) The bacterial distribution along the channel at 1 h (light gray), 3 h (gray), and 6 h (dark gray) after setting the source: *Left plot* – wild type cells with no effectors at the source. *Middle plot* – non-chemotactic cells (Δ*cheY*Δ*cheZ*) with MeAsp (1mM) at the source. *Right plot* – non-chemotactic cells (Δ*cheY*Δ*cheZ*) with low pH (4.5) at the source.

Supplementary: **Figure S2**

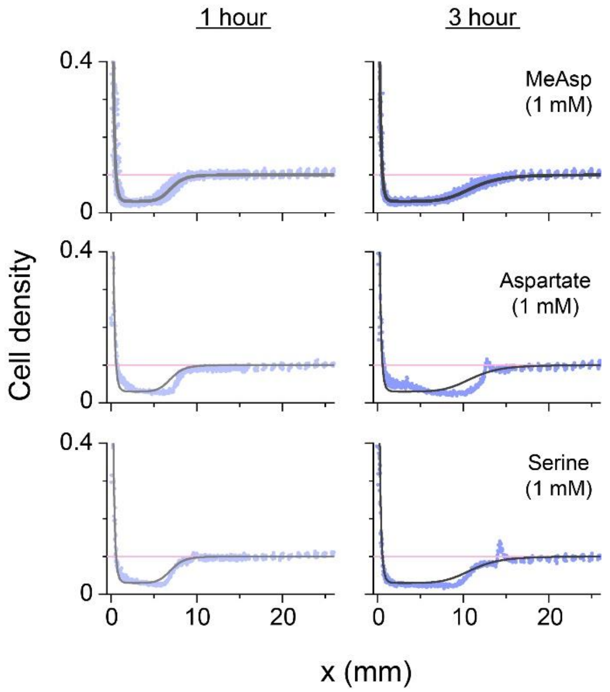

Fig. S2. The bacterial responses to serine and aspartate**.**

The distribution of wild-type bacteria 1 or 3 hours (as labeled) after adding MeAsp, aspartate, or serine to the source, as labeled. To allow for direct comparison, the lines describing the behavior with MeAsp (top plot) are also shown in the middle and bottom plots.

Supplementary: **Figure S3**

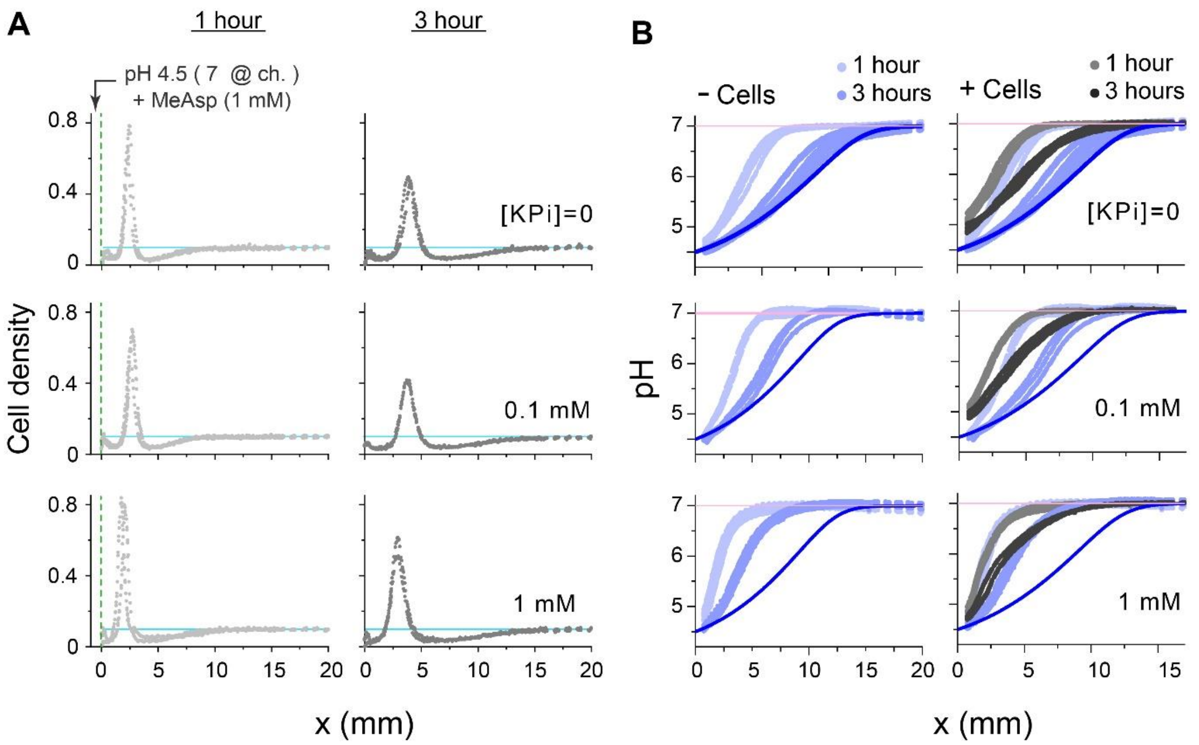

Fig. S3. The effect of buffering capacity on the bacterial redistribution and pH profiles.

(**A**) The bacterial redistribution with low pH (4.5) and MeAsp (1 mM) at the source for various concentrations of the phosphate buffer, as labeled. Profiles are shown for 1 (left) and 3 (right) hours after the introduction of the source.

(**B**) The pH profiles measured 1 and 3 hours after the source was introduced (as labeled) for experiments that were done in the absence (blue symbols) or presence (gray symbols) of non-fluorescent bacteria in the channel. The blue line marks the expected distribution should the pH expansion follow a generic diffusion pattern with $D = 0.75 \cdot 10^{-5}$ cm^2/sec (the value used for MeAsp). Note that in the presence of cells the pH near the source tends to be somewhat elevated. These profiles where measured by adding uniform distribution of Fluorescein along the channel (10 µM) and following its fluorescence intensity profile over time, which was independently calibrated against the pH in bulk samples.

Supplementary: **Figure S4**

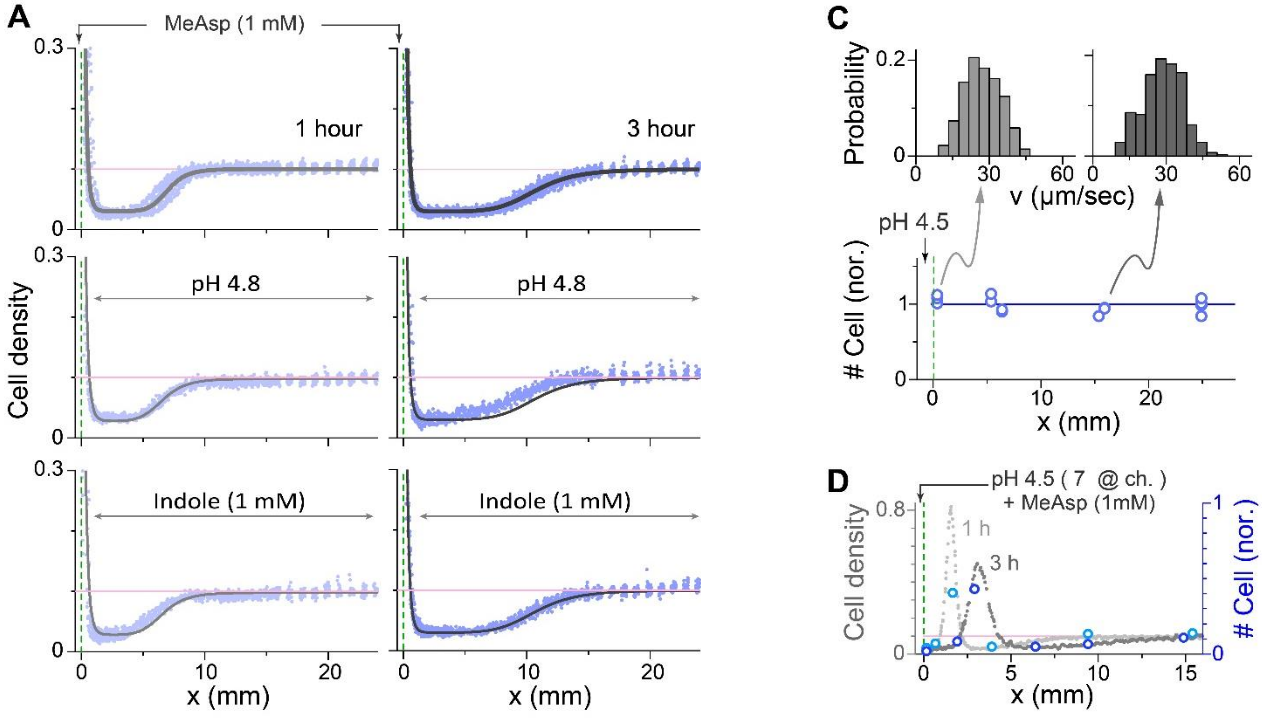

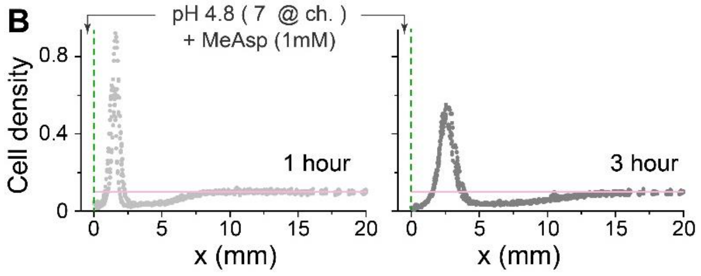

Fig. S4. The inherent effect of the repellents on the bacteria. (**A**) The bacterial response to MeAsp (1 mM) at the source, measured in the absence of background (upper plot), or with uniform background of either low-pH (4.8, middle plot) or indole (1mM, lower plot). To allow for direct comparison, the lines describing the behavior in the absence of background (upper plot) are also shown in the lower plots. (**B**) The bacterial redistribution 1 and 3 hours after the introduction of a low pH (4.8) and MeAsp (1 mM) to the source. (**C**) *Lower plot* – The distribution of Δ(*tar tsr*) cells 3 hours after setting the pH at the source to 4.5, estimated by counting the number of cells per image along the channel. *Upper plot* – The histograms of cell's velocity near the source (left, x < 0.5 mm) or away from the source (right, x > 5 mm). (**D**) The bacterial density profile 1 and 3 hours after the introduction of low pH (4.5) and MeAsp (1 mM) to the source, estimated by the fluorescence intensity, or by cell counting.

Several lines of evidence demonstrated that setting the pH at the source to 4.5 did not have a significant effect on the bacterial physiology or directly distort the results (see also main text):

(**a**) The bacterial accumulation generically observed with pH 4.5 and MeAsp at the source was similarly observed with pH 4.8 at the source (Fig. S4B). Note that pH 4.8 did not affect the chemotactic ability of the cells nor on their inherent fluorescence.

(**b**) The bacterial accumulation shifted away from the pH-4.5 source as the MeAsp concentration at the source was increased (Fig. 2A, inset), indicating that the bacterial accumulation is not directly related to any harmful effect of the pH near the source.

(**c**) The distribution of Δ(*tar tsr*) cells along the channel, indicated by their fluorescence profile, was not affected by setting the pH source to 4.5 (Fig. 4A, inset). Moreover, not only the fluorescence profile, but also the number of cells per image along the channel was not affected (Fig. S4C, lower plot). Taken together, we can also conclude that the intrinsic fluorescence intensity of the cells was not significantly affected, even close to the source. Finally, the swimming speed of the bacteria was also not affected near the source (Fig. S4C, upper plots).

(**d**) The bacterial accumulation created by combination of pH 4.5 and MeAsp (1 mM) at the source could be also observed by counting the number of cells per image along the channel, and was similar to that observed by recording the fluorescence profile (Fig. S4D, blue and grey symbols, respectively). Note, that the cell count at the peak is significantly underestimated owing to the difficulty of resolving single cells at the high cell density environment.

(**e**) Clearly, setting the pH at the source to 4.5 clearly did not prevent ΔTsr cells from approaching and accumulating at the source (Fig. 4A, red symbols). These cells were clearly swimming very close to the source.

Supplementary: **Figure S5**

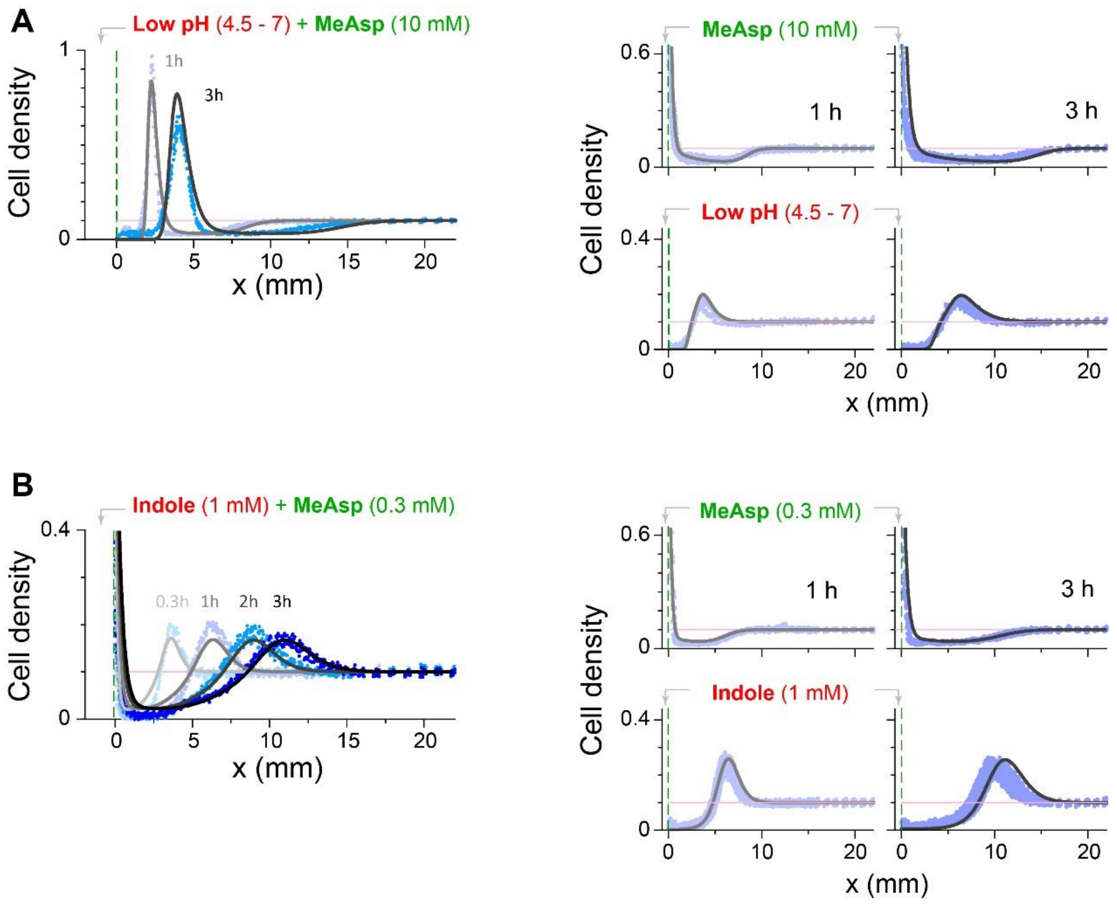

Fig. S5. A comparison between the simulation's outputs (gray lines) and the observed bacterial behaviors (blue symbols).

(**A**) The bacterial response with low pH and MeAsp at the source (left plot; data was taken from Fig. 2B) and the responses to the individual effectors (right plots).

(**B**) The bacterial response with indole and MeAsp at the source (left plot; data was taken from Fig. 2D) and the responses to the individual effectors (right plots).

The parameters used in the simulations:

For the MeAsp force, $\alpha = 25\cdot10^{-3}$ cm/sec, $k_{on} = 20$ mM, $k_{off} = 20$ μM and $D_{MeAsp} = 0.75\cdot10^{-5}$ cm^2/sec.

For the low pH force, $\alpha = 5\cdot10^{-3}\cdot f_{pH}$ where $f_{pH} = 1 + 9 / [1 + (10^{-5.4}/ C_{H+}(x,t))^2]$, phenomenologically accounts for the expected enhancement of the response near the source where the pH is low (see text), and $[C_{H+}] = M$. $D_{pH} = 0.2\cdot10^{-5}$ cm^2/sec, $k_{on} = 10^{-7}$ M, $k_{off} = 10^{-3}$ M.

For the indole force, we used the same parameters as detailed in Fig. S6 below. D_{cells} was set to $0.8\cdot10^{-5}$ cm^2/sec in the case of the response to repellents alone (indole, pH) while it was set to $0.4\cdot10^{-5}$ cm^2/sec in all other cases.

Supplementary: **Figure S6**

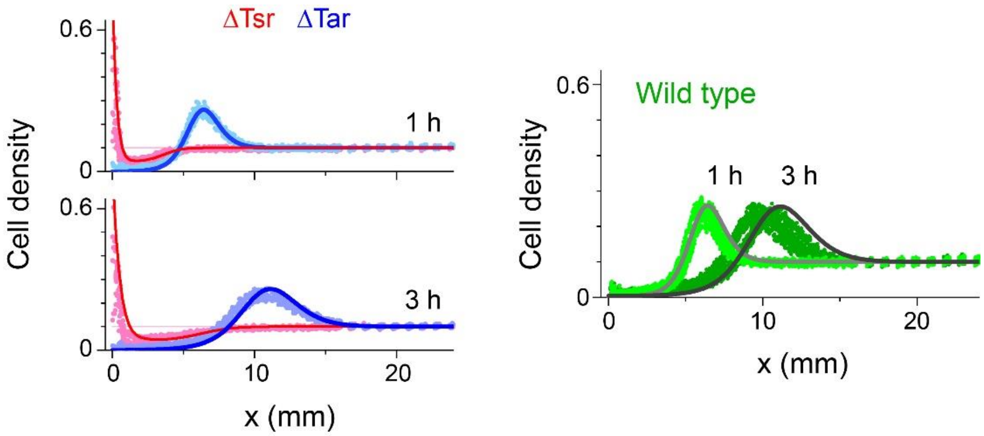

Fig. S6. Analysis of the indole force as a product of an attractive and a repulsive component.

The response of ΔTar (left plot, blue symbols), ΔTsr (left plot, red symbols), and wild type (right plot) cells to indole (1mM). The force applied in the simulations to describe the behavior of the wild type cells was the sum of the individual forces used to describe the mutants.

The parameters used in the simulations:

For the indole-repulsive force (ΔTar data), k_{on} = 20 μM, k_{off} = 3 mM, and α = $20 \cdot 10^{-3}$ cm/sec.
For the indole-attractive force (ΔTsr data), k_{on} = 20 mM, k_{off} = 0.2 mM, and α = $25 \cdot 10^{-3} \cdot f_{ind}$,
where $f_{ind} = [1 + (3 \cdot 10^{-4} / C_{indole}(x, t))^2]^{-1}$ accounts for the limited range of influence observed with the ΔTsr cells, and [C_{indole}]=M. The indole diffusion constant was set to $0.8 \cdot 10^{-5}$ cm^2/sec.

Supplementary: **Figure S7**

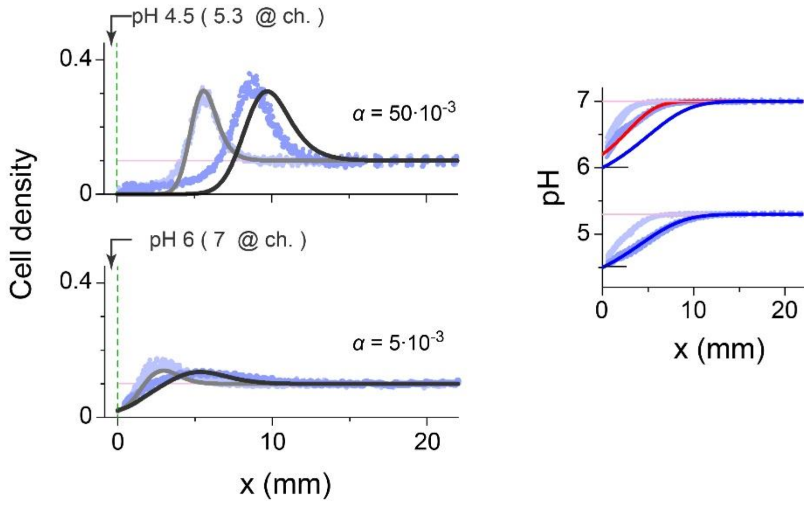

Fig. S7. Quantifying the enhancement of the pH response at low pH.

Left plots – The response of ΔTar cells to a low-pH source. In the experiment presented in the upper plot, the source pH was 4.5 while the bulk of the channel was set to 5.3. In the experiment presented in the lower plot, the source pH was 6 while the bulk of the channel was set to 7. Data is shown for 1 (light-blue symbols) and 3 (darker-blue symbols) hours. The outputs of the corresponding simulations are also shown (gray lines). Notably, to account for data, the value of *α* had to be approximately 10-fold higher at the lower pH range compared with the higher pH range (as labeled). The rest of the parameters were similar to those used in Fig. S5.

Right plot – The corresponding pH profiles (blue symbols). Simple diffusion approximations are also shown, using $D_{pH} = 0.9 \cdot 10^{-5}$ cm^2/sec (blue line) or $D_{pH} = 0.4 \cdot 10^{-5}$ cm^2/sec (red line). These approximated diffusion coefficients were used in the simulations.

Supplementary: **Figure S8**

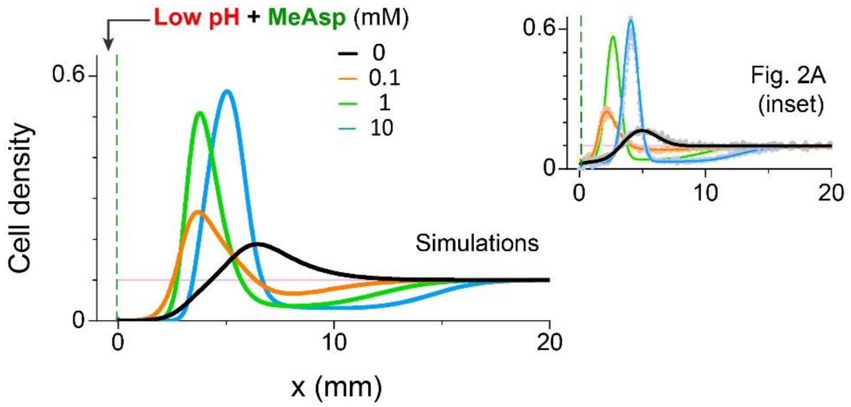

Fig. S8. An interference of the attractant in the repellent force can account for the bacterial response to a source of low pH and varying MeAsp concentrations.

In the inset, the data shown in Fig. 2A (inset) is replotted. The simulations shown in the main figure were carried out using the same parameters used before (Fig S5), but with a modified enhancement function for the pH response that phenomenologically accounts for the dependence on MeAsp, demonstrated in Fig 4B. The modified enhancement function was:

$$\alpha = 5 \cdot 10^{-3} \cdot f_{MeAsp-pH}$$

$$where\ \ f_{MeAsp-pH} = 1 + \frac{3}{1 + (10^{-5.3}/C_{H^+}(x,t))^{2.5}} \cdot \frac{1}{1 + 1.5/(1 + (0.5/C_{MeAsp}(x,t))^2)}$$

where $[C_{MeAsp}] = mM$ and $[C_{H+}] = M$.
